# Cone Beam Computed Tomography Evaluation of the Periapical Status of Nonvital Tooth with Open Apex Obturated with Mineral Trioxide Aggregate: A Case Report

**DOI:** 10.1155/2013/714585

**Published:** 2013-03-31

**Authors:** Vijay Shekhar, K. Shashikala

**Affiliations:** Department of Conservative Dentistry and Endodontics, D.A.P.M.R.V. Dental College and Hospital, Bangalore, Karnataka 560078, India

## Abstract

Management of a tooth with open apex is a challenge to the dental practitioners. Evaluation of the periapical healing is required in such cases by radiographic techniques. The objective of this paper was to assess the healing of a periapical lesion in a non-vital tooth with open apex treated with mineral trioxide aggregate (MTA) obturation using cone beam computed tomography (CBCT). The endodontic treatment of a fractured non-vital discolored maxillary left lateral incisor with an open apex was done with MTA obturation. The clinical and radiographic followup done regularly showed that the tooth was clinically asymptomatic and that the size of the periapical lesion observed by intraoral periapical (IOPA) radiographs and CBCT was decreased remarkably after two years. CBCT and IOPA radiographs were found to be useful radiographic tools to assess the healing of a large periapical lesion in a non-vital tooth with open apex managed by MTA obturation.

## 1. Introduction

Periapical lesion of endodontic origin is one of the most frequently occurring pathologic conditions found in the alveolar bone. It represents an inflammatory response to bacterial infection of the root canals. Periapical lesions may progress from the inflammation of the periodontal ligament around the root apex (apical periodontitis) into a variety of pathologic conditions, like apical abscess, periapical granuloma, and radicular cyst. Besides the possible clinical signs and symptoms associated with the periapical lesions, there are changes in the mineralization and structure of the periradicular bone which results in resorption. This can be visualized by various radiographic techniques as periapical radiolucencies. Their management is initially done with nonsurgical endodontic treatment. Surgical intervention is recommended when nonsurgical procedures have been unable to resolve the periapical lesions. Studies have reported high success rate (upto 94%) of healing of periapical lesions following non-surgical endodontic therapy [1].

Complete asepsis and three-dimensional obturation of the root-canal system is essential for long-term endodontic success [2, 3]. Cessation of root development caused by trauma or pulpal disease presents both endodontic and restorative challenges. The thin fragile root-canal dentin walls may be too weak to withstand the normal forces of mastication, making them prone to fracture [3, 4]. The divergent apical architecture (blunderbuss canal) and absence of normal apical constriction of root canal make complete debridement, canal disinfection, and control of obturation material nearly impossible [3–7]. Occasionally, the filling is inadequate or the filling materials extrude beyond apex into periradicular tissues, which may have harmful effect on the prognosis of endodontic treatment. The use of biomaterials has been advocated to encourage periapical regeneration and prevent complications if overfilling occurs [8, 9]. 

Apexification with calcium hydroxide was the treatment of choice in necrotic teeth with open apex in the last decades [3, 4, 6–11]. The long-term use of calcium hydroxide had several disadvantages, including multiple appointments, possible recontamination of the root canal during the treatment period, and increased brittleness of the root dentin [2–4, 8, 9]. The new treatment concept involves eliminating the gutta-percha obturation step after apexification. MTA, placed in an apexification technique, could become the sole obturation material, preserving and restoring the open apex [4].

Mineral trioxide aggregate (MTA; ProRoot, Dentsply, Tulsa, OK, USA) has been proposed as obturation material as it can completely seal the root-canal system [1, 4, 5]. According to the manufacturer, MTA is a mixture of three powder ingredients: Portland cement, bismuth oxide, and gypsum. When mixed with sterile water, hydration reaction occurs and MTA sets, even in the presence of moisture. The pH of MTA increases from 10 to 12.5, three hours after mixing. It is assumed that in a high pH environment, calcium ions, that are released from MTA, react with phosphates in the tissue fluid to form hydroxyapatite. This would explain its favorable sealing ability and biocompatibility [1, 10, 11]. MTA has shown potential as a root-end filling material, root/furcal perforations sealing material, and a direct pulp capping agent after pulp exposure, pulpotomy agent, and root-canal filling material in teeth with complete and incomplete root development. Despite its outstanding tissue biocompatibility, MTA has few disadvantages, which include delayed setting time, poor handling characteristics, unpredictable antibacterial effects, and high cost [1].

It has long been debated whether bone healing after endodontic treatment is ongoing or whether the treatment effort has been futile. The endodontic treatment is considered successful when there is absence of periapical radiolucency at periapex of endodontically treated roots. The newer diagnostic method such as cone beam computed tomography (CBCT) is considered as a useful diagnostic tool for assessing periapical healing by measuring true size of the periapical lesion three-dimensionally, which is mostly underestimated by intraoral periapical (IOPA) radiographs [12–15]. 

CBCT is one of the latest and most advanced types of digital radiography. Here, an object is exposed to multiple cone-shaped beams that travel 360° around the patient with the motion center placed in the area of interest and the X-ray detector on the opposite side of the circle. Later, serial sagittal, coronal, and axial section images are obtained, allowing the clinician to visualize morphologic features and pathologies from different three-dimensional perspectives eliminating the superimposition of anatomical structures. For endodontic purposes, the limited volume or focused CBCT scanners are used which capture small volumes of data encompassing just 3-4 individual teeth. CBCT may also be useful in the evaluation of the root-canal anatomy, canal preparation, obturation, retreatment, coronal microleakage, and periapical lesions, including their relatively true size, extent, nature, and position.

The following case report describes evaluation of healing of a large periapical lesion in an asymptomatic non-vital left maxillary lateral incisor with an open apex treated with MTA obturation using CBCT and IOPA radiographs over a period of two years. 

## 2. Case Report

A 24-year-old healthy female patient was reported with the chief complaint of broken left upper front teeth. Patient had history of fall 15 years back which resulted in fracture of the crowns and occasional mild pain in left upper front teeth since then, for which she took painkillers prescribed by a local doctor to provide symptomatic relief. There was no history of discharge or swelling. The patient came for definite treatment six months back.

**Figure 1:**
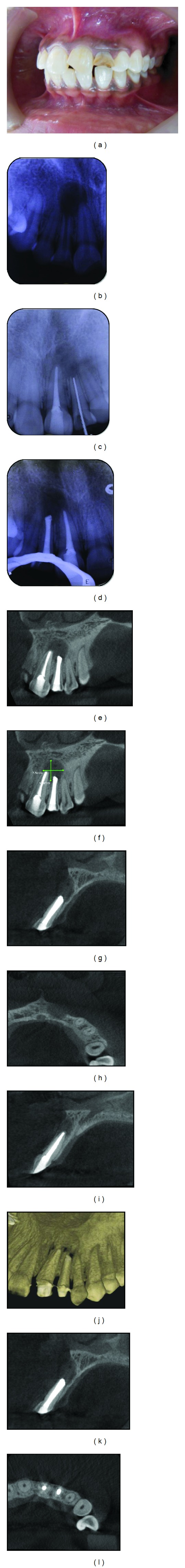
(a) Preoperative photo showing crown fracture and discoloration in left maxillary lateral incisor. (b) IOPA radiograph showing well-defined radiolucency and open apex. (c) Working length IOPA radiograph. (d) IOPA radiograph showing MTA obturation. (e) Coronal plane CBCT image showing size of periapical lesion immediately after MTA obturation. (f) Coronal plane CBCT image showing size of periapical lesion; dimensions 9.8 mm/7.5 mm. (g) Sagittal plane CBCT image showing size of periapical lesion immediately after MTA obturation. (h) Axial plane CBCT image showing size of periapical lesion immediately after MTA obturation. (i) Sagittal plane CBCT image showing size of periapical lesion immediately after endodontic treatment in tooth 21. (j) Three-dimensional CBCT image of periapical lesion immediately after MTA obturation. (k) Sagittal plane CBCT image showing status of MTA obturation. (l) Axial plane CBCT image showing status of MTA obturation.

On clinical examination, Ellis class 2 fracture was noted in teeth 21 and 22 (Figure 1(a)). Vitality (heat, cold and electric) tests revealed that both teeth were non-vital. Tooth 22 was discolored. There were no signs of maxillofacial fracture or swelling or mobility. The soft tissue examination was noncontributory. Radiographic examination with IOPA radiograph revealed a well-defined radiolucency with respect to the apex of both teeth and a wide open apex in tooth 22 (Figure 1(b)).

Based on the clinical and radiographic findings, a diagnosis of periapical granuloma involving teeth 21 and 22 was established. A treatment plan involving endodontic therapy was proposed, with MTA obturation in tooth 22, followed by ceramic crowns.

The endodontic treatment was done under rubber dam isolation. First, access opening was done, followed by working length determination (Figure 1(c)). Cleaning and shaping were done by circumferential filing in tooth 22. Obturation of tooth 21 was done with conventional lateral condensation of gutta-percha. In tooth 22, calcium hydroxide dressing was placed in the first appointment, which was removed one week later, with alternate sodium hypochlorite and saline irrigation. The canals were thoroughly dried with paper points. The ProRoot gray MTA was mixed to a paste consistency with sterile water and delivered to the canal using Messing gun. A plugger was used to condense MTA homogeneously into the canal (Figure 1(d)). A moist cotton pellet was sealed inside for setting of MTA. It was noteworthy that there was inadvertent extrusion of MTA into the periapical region during its compaction. 

**Figure 2:**
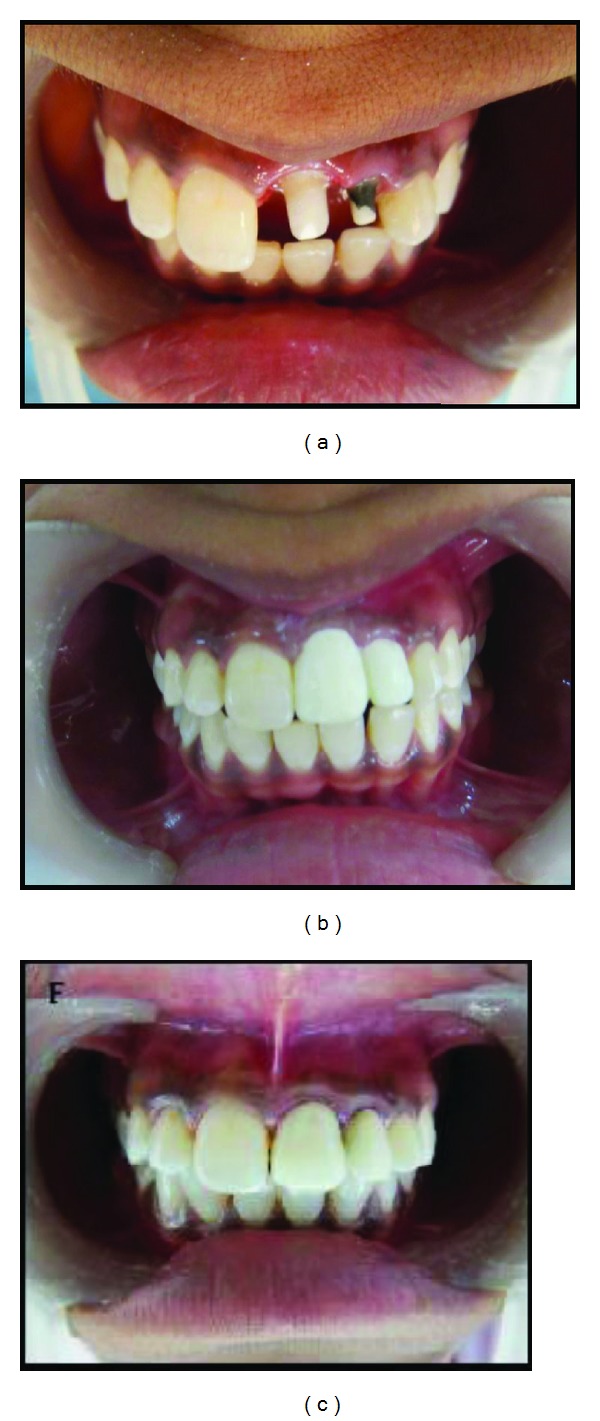
(a) Build-up with composite and tooth preparation. (b) Temporization with acrylic crowns. (c) Permanent cementation of PFM crowns.

The composite core build-up and tooth preparation was done on both teeth for ceramic crowns in the next appointment (Figure 2(a)). Temporization was done with acrylic crowns (Figure 2(b)). The occlusion was carefully checked and adjusted. Permanent crowns were not placed immediately as they would have interfered with the planned postoperative followup of the healing of periapical lesion using CBCT. The patient was given instructions to avoid any activities that could lead to trauma to the teeth and was recalled for followup. The permanent crowns, that is, porcelain fused-to-metal (PFM) crowns, were cemented on both teeth six months after the endodontic treatment and followup with CBCT (Figure 2(c)).

**Figure 3:**
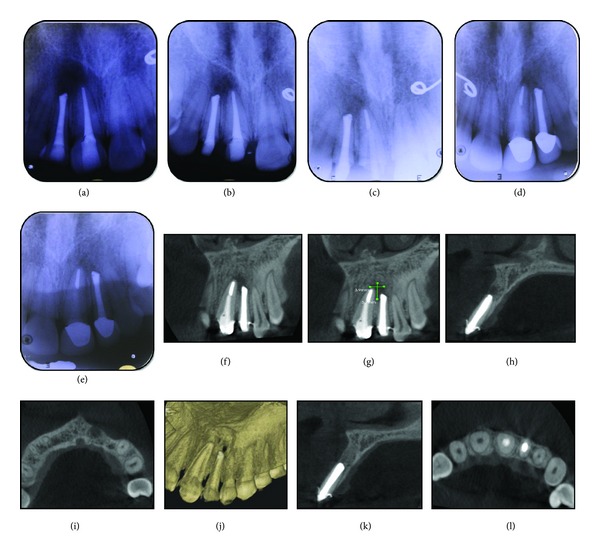
(a) IOPA radiograph at one month. (b) IOPA radiograph at three months. (c) IOPA radiograph at six months. (d) IOPA radiograph at one year. (e) IOPA radiograph at two-year followup. (f) Coronal plane CBCT image showing decreased size of periapical lesion at six months. (g) Coronal plane CBCT image showing measurement of periapical lesion; dimensions 5.7 mm/3.9 mm. (h) Sagittal plane CBCT image showing decrease in size of periapical lesion at six months. (i) Axial plane CBCT image showing decrease in size of periapical lesion at six months. (j) Three-dimensional CBCT image showing periapical lesion six months. (k) Sagittal plane CBCT image showing status of MTA obturation at six months. (l) Axial plane CBCT image showing status of root-canal filling at six months.

The postoperative evaluation of the periapical lesion was done clinically as well as radiographically, with both IOPA radiographs and CBCT. The IOPA radiographic evaluation revealed a decreasing size of the periapical radiolucency at each visit after one month, three months, six months, one year, and two years (Figures 3(a)–3(e)). The follow-up radiograph taken two years after treatment revealed that the periapical radiolucency was almost unnoticeable, in relation to both teeth. 

CBCT examination was done immediately after MTA obturation (Figures 1(e)–1(l)) using limited field of view CBCT unit (9000 3D, KODAK, USA). The second CBCT evaluation done after six months revealed a decrease in the size of periapical radiolucency in all the three planes—coronal, sagittal, and axial and in three-dimensional reconstruction images (Figures 3(f)–3(j)). The status of MTA obturation as evaluated with CBCT revealed a well compacted filling up to the apex with no voids, even after six months (Figures 3(k)-3(l)). The patient when observed clinically after two years was asymptomatic and had no complaint of discomfort. The patient is scheduled to come for follow-up yearly in the department.

## 3. Discussion

Bacterial infection of the dental pulp may lead to periapical lesion. Its treatment involves eradicating or substantially reducing the number of root-canal pathogens and preventing reinfection by three-dimensional obturation. When endodontic treatment is done properly, healing of the periapical lesion occurs with hard tissue regeneration, which is characterized by gradual resolution of the periapical radiolucency [1].

In the present case, the radiographic evaluation of the periapical lesion after two years revealed an excellent treatment outcome which is comparable with similar previously reported cases of successful MTA apexification in necrotic open apex teeth with periradicular lesions. In such teeth, the outcome of conventional gutta-percha fillings would be doubtful, while MTA has the potential to provide an effective seal, even in the presence of moisture such as in a blunderbuss root canal [5]. Furthermore, several studies have confirmed the superior biocompatibility of MTA as a root-end filling material for the periodontal tissues and the regeneration of periradicular tissues to an almost normal condition [8].

When treating non-vital teeth, a main issue is eliminating bacteria from the root canal system. In this case, in order to limit bacterial infection before obturation with MTA, irrigation with sodium hypochlorite was done and short-term intracanal calcium hydroxide medication was placed within the canal for one week. The rationale was to enhance the difficult task of debriding the canal system with an open apex [6]. Researchers showed that the remains of calcium hydroxide on the dentinal walls had no significant effect on MTA microleakage. Others have suggested that the combination of MTA and calcium hydroxide in apexification procedures may favorably influence the regeneration of the periodontium. 

In necrotic teeth with open apex, the absence of apical constriction complicates the adaptation of MTA to root canal. A hand condensation technique was used in this case to overcome this difficulty. This technique resulted in improved adaptation and fewer voids than the thermoplasticised gutta-percha techniques used traditionally. Due to the physical and handling properties of MTA, this material is more likely to extrude apically in an open apex. When the extruded root-canal filling material contacts periradicular tissues, reactions like mild to severe inflammation, allergic reactions, and neurotoxic effects may occur. Gutta-percha and calcium hydroxide are the most common materials associated with these complications. Conversely, the extrusion of MTA root-canal filling biomaterial into a periradicular lesion does not produce complications, which was confirmed with CBCT in this case. Recently, the biological basis for the favorable properties of MTA has been attributed to the production of hydroxyapatite. Furthermore, the deposition of cellular cementum adjacent to MTA as a root filling has been demonstrated. The case reported here demonstrated that when MTA is used as an obturation material in necrotic tooth with open apex, the canal can be effectively sealed. It has an added advantage of speed of completion of therapy and periapical healing that follows [7].

Followup is of utmost importance to observe success of the treatment. Various studies have recommended that followup should be performed for a minimum of one year. In this study, followup was done for two years and the patient is scheduled for further recall visits yearly. The clinical and radiographic examinations by CBCT and IOPA radiographs done at each visit revealed a remarkable decrease in the size of the periapical radiolucency.

CBCT has enabled the early and accurate detection of periapical lesion and the spatial relationship of these lesions to important anatomic landmarks. A major advantage of limited (small field of view) CBCT scanner, which was used in this case, is the relatively low-effective radiation dose the patient is exposed to. It yields radiation doses similar to that from two to three intraoral radiographs. However, the difference in information is considerably larger as compared to periapical radiography. The use of CBCT has also provided new capabilities for assessment of the periapical healing. In this case, an immediate postoperative radiograph was taken along with a CBCT scan, which showed the extent of MTA extrusion into periapical area as well as the extent and size of the periapical lesion. The latter has an advantage of evaluation using the same radiographic criteria at each appointment postoperatively. The IOPA radiographs and CBCT images show how distinctly different they are and how futile it is to compare one with the other. Our results also show how superior the CBCT images are, particularly in evaluation of status of MTA obturation and periapical healing in traumatized teeth with open apex. 

## 4. Conclusion

This paper revealed that CBCT is a better and more accurate radiographic tool as compared to IOPA radiographs to assess the healing of a large periapical lesion associated with a non-vital tooth with open apex. Also, MTA obturation in such a case was found to result in favourable treatment outcome two years postoperatively.
